# The Piston Slap Force Reconstruction of Diesel Engine Using WOA-VMD and Deconvolution

**DOI:** 10.3390/s24123833

**Published:** 2024-06-13

**Authors:** Shigong Fan, Yixi Cai, Yunxi Shi, Zongzhen Zhang

**Affiliations:** 1 School of Automotive and Traffic Engineering, Jiangsu University, Zhenjiang 212013, China; qc001@ujs.edu.cn (Y.C.); shiyunxi880527@126.com (Y.S.); 2 College of Mechanical and Electronic Engineering, Shandong University of Science and Technology, Qingdao 266000, China; zhzz18@126.com

**Keywords:** piston slap force reconstruction, impulse function, variational mode decomposition, WOA, deconvolution, regulation method

## Abstract

In a diesel engine, piston slap commonly occurs concurrently with fuel combustion and serves as the main source of excitation. Although combustion pressure can be measured using sensors, determining the slap force is difficult without conducting tests. In this study, we propose a method to identify the slap force of the piston to solve this difficult problem. The traditional VMD algorithm easily receives noise interference, which affects the value of parameter combination [*k*, *α*] and thus affects the extraction accuracy of the algorithm. First, we obtain the transfer function between the incentive and vibration response through percussion tests. Secondly, a variational modal decomposition method based on whale algorithm optimization is used to separate the slap response from the surface acceleration of the block. Finally, we calculated the slap force using the deconvolution method. Deconvolution is a typical inverse problem of mathematics, often prone to ill-conditioning, and the singular value decomposition and regularization method is used to overcome this flaw and improve accuracy. The proposed method provides an important means to evaluate the angular distribution of the slap force, identify the shock positions on the piston liner, and determine the peak value of the waveform which helps us analyze the vibration characteristics of the piston and optimize the structural design of the engine.

## 1. Introduction

The diesel engine offers a comfortable driving experience, particularly for trucks and agricultural machinery, because of its high torque at low speeds. Manufacturers consider the acoustic optimization of diesel engines as the most critical task, in addition to reducing fuel consumption and exhaust gas emissions. The major sources of noise in diesel engines include combustion noise, mechanical noise, and aerodynamic noise. One of the mechanisms of the generated noise in a reciprocating piston engine is the motion of the piston because its clearance is perpendicular to its normal running direction. The gas and inertial forces, along with the connecting rod angle and necessary installation clearance, inevitably result in the transverse motion of the piston. As the piston and the crank mechanism reciprocate through intake, compression, expansion, and exhaust strokes, a connecting rod reaction force (Fa) acts on the piston with alternating direction. This reaction force contains a lateral component (Fs), which causes the piston to move across the clearance and impact the cylinder wall. When the piston contacts the cylinder wall, it generates impacts that excite structure-borne noise within the engine structure.

**Figure 1:**
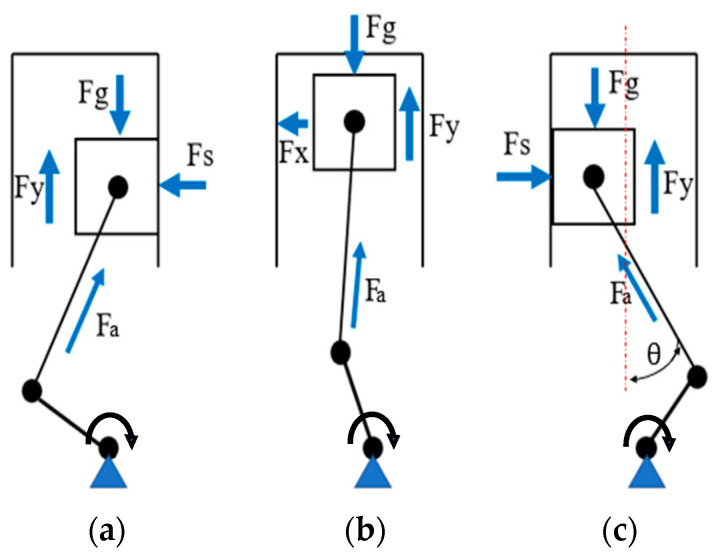
The motion and forces of piston at different strokes; (**a**) before TDC; (**b**) during TDC; (**c**) after TDC.

The generation of slap noise depends on various factors, including the piston–liner gap, type of lubricant used, piston pin offsetting [1], and piston geometry [2]. The analysis of lateral and rotary motion of the piston within the gap between the cylinder liner and piston has been conducted. A model that can predict the forces and response of the engine block resulting from slap has been discussed. Parameters, such as mass, spring, and damping constant, have been predicted using a vibrational mobility model [3]. Several methods have been employed to estimate the impact forces. The finite element method (FEM) has been used to analyze the impact force [4,5]. Furthermore, a two-dimensional lumped parameter model has been proposed to calculate the impact force [6]. Meanwhile, the numerical simulation results are compared with measured vibration velocity level at engine block surfaces to verify the proposed model. As shown in Figure 1, the piston moves in three directions, namely vertical, transverse, and rotating directions. The transverse and rotating directions are called the secondary movement of the piston, which has a great impact on the impact of the piston. 

In engineering practice, obtaining the actual slap force through experimental testing is a challenging task. Thus, most methods rely on numerical simulations to estimate the slap, which is extremely valuable in recovering the piston slap force from external structural vibration measurements on an engine block. A good method for identifying the cylinder pressure is applying an inverse filter of a specific structure to the vibration signal in an IC engine [7,8,9,10], which is based on angular sampling and cyclic signal processing, shedding light on the condition monitoring of internal combustion engines.

In this study, we conducted three-part tasks to recover the piston slap. In Section 2, the vibration signal produced by the slap force is separated from the total response using a WOA-based VMD method. Then, the impulse response function is acquired between the slap and the structure vibration of the cylinder block through hammer testing. Finally, a deconvolution method was used to reconstruct the slap force. 

## 2. Materials and Methods

### 2.1. Piston Slap Response Extraction by VMD Method

Variational mode decomposition is a time-frequency analysis method for processing nonlinear and nonstationary signals, which overcomes the disadvantage of frequency aliasing of empirical mode decomposition [11].

In order to obtain the most favorable degradation results, we use the whale optimization algorithm (WOA) [12] to optimize parameter combinations and reduce the significant impact of parameter selection on the results of variational mode decomposition (VMD). The assumption of this method is that each intrinsic mode function (IMF) has a finite bandwidth and different center frequencies. The conversion process solves the problem of variation in order to minimize the total estimated bandwidth of IMF [13]. Each IMF is demodulated to its corresponding baseband, and each IMF and its corresponding center frequency is derived.

Suppose each component has a limited bandwidth centered around a certain madness that is constantly updated during the separation process. Therefore, the original signal $f\left(t\right)$ can be divided into modal components $u_{k}\left(t\right)$ with a central frequency $\omega_{k}\left(t\right)$, where *k* represents the standard quota [13,14,15]. The modal degradation algorithm of fractional order constructs and solves the variation multiple function. Equations (1) and (2) can be used to describe the relationship between the impact forces on the inner wall and the surface vibration of the object.

(1) $$a\left(t\right)=a_{s}\left(t\right)+a_{c}\left(t\right)$$

(2) $$a_{s}\left(t\right)=h\left(t\right)*f_{s}\left(t\right)$$

(1) The Hilbert transform is used to derive the analytical signal and its unilateral spectrum for each modal function $u_{k}\left(t\right)$, as shown in Equation (3).

(3) $$\left(\delta \left(t\right)+\frac{j}{\pi t}\right)*u_{k}\left(t\right)$$

where $\delta \left(t\right)$ is an impact function.

(2) Each mode is modulated to its corresponding baseband because the estimated center frequency $e^{-j\omega_{k}t}$ of each modal analysis signal is mixed, as shown in Equation (4).

(4) $$\left(\left(\delta \left(t\right)+\frac{j}{\pi t}\right)*u_{k}\left(t\right)\right)e^{-j\omega_{k}t}$$

(3) The bandwidth of each mode signal and the square $L^{2}$ norm of the given demodulation signal gradient are calculated. The objective function is shown in Equation (5).

(5) $$\left\{\begin{array}{l} \underset{\left\{u_{k}\right\},\left\{\omega_{k}\right\}}{\min}\left\{\sum_{k=1}^{K}{\left\|\partial_{t}\left(\left(\delta \left(t\right)+\frac{j}{\pi t}\right)*u_{k}\left(t\right)\right)e^{-j\omega_{k}t}\right\|}_{2}^{2}\right\}\\ s.t.\sum_{k=1}^{K}u_{k}=f \end{array}\right.$$

where $\left\{\left.u_{k}\right\}\right.=\left\{\left.u_{1},\ldots ,u_{k}\right\}\right.$ is the IMF components, and $\left\{\left.\omega_{k}\right\}\right.=\left\{\left.\omega_{1},\ldots ,\omega_{k}\right\}\right.$ is the central frequency of each component.

(4) The Lagrangian multiplication operator $\lambda \left(t\right)$ and second-order penalty factor *a* are introduced to transform the objective function into an unconstrained variational objective function. *a* ensures the fidelity of the reconstructed signal in the presence of Gaussian noise, while $\lambda \left(t\right)$ strictly enforces constraints. The extended Lagrangian expression is shown in Equation (6).

(6) $$\begin{array}{l} L\left(\left\{\left.u_{k}\right\},\left\{\left.\omega_{k}\right\},\right.\lambda \right.\right)=\alpha \sum_{k}{\left\|\partial_{t}\left(\left(\delta (t)+\frac{j}{\pi t}\right)*u_{k}(t)\right)e^{-j\omega_{k}t}\right\|}_{2}^{2}+{\left\|f(t)-\sum_{k}u_{k}(t)\right\|}_{2}^{2}\\ +\langle \lambda \left(t\right),f(t)-\sum_{k}u_{k}(t)\rangle \end{array}$$

The alternating direction method of the multiplier is used for iterative sub optimization to handle sub signals and center frequencies. Subsequently, Equation (6) is transformed into the frequency domain using the Parseval/Plancherel Fourier Isometric Transform:

(7) $$H=\left[\begin{array}{ccccccc} h\left(1\right) & h\left(2\right) & h\left(3\right) & \cdots & h\left(k\right) & \cdots & h\left(m\right)\\ & h\left(1\right) & h\left(2\right) & \cdots & h\left(k-1\right) & \cdots & h\left(m-1\right)\\ & & h\left(1\right) & \cdots & h\left(k-2\right) & \cdots & h\left(m-2\right)\\ & & & \ddots & \vdots & \vdots & \vdots \\ 0 & & & & h\left(1\right) & \cdots & h\left(m-k\right) \end{array}\begin{array}{cccc} & & & 0\\ h\left(m\right) & & & \\ h\left(m-1\right) & h\left(m\right) & & \\ \vdots & \vdots & \ddots & \\ h\left(m-k+1\right) & h\left(m-k+2\right) & \cdots & h\left(m\right) \end{array}\right]$$

The first variable of positive frequency is eliminated, and two optimization problems can be solved as follows:

(8) $${\hat{u}}_{k}^{n+1}(\omega )=\frac{\hat{f}(\omega )-\sum_{i\neq k}{\hat{u}}_{i}(\omega )+\frac{\hat{\lambda }(\omega )}{2}}{1+2\alpha {\left(\omega -\omega_{k}\right)}^{2}}$$

The same solving process is used, and the center frequency solving problem is transformed into a frequency domain.

(9) $$\omega_{k}^{n+1}=\underset{\omega_{k}}{\arg\min}\left\{\left.\int_{0}^{\infty }{\left(\omega -\omega_{k}\right)}^{2}{\left|{\hat{u}}_{k}(\omega )\right|}^{2}d\omega \right\}\right.$$

Deriving the updated formula for center frequency from Equation (10):

(10) $$\omega_{k}^{n+1}=\frac{\int_{0}^{\infty }\omega {\left|{\hat{u}}_{k}(\omega )\right|}^{2}d\omega }{\int_{0}^{\infty }{\left|{\hat{u}}_{k}(\omega )\right|}^{2}d\omega }$$

The iterative process continues until the convergence criterion is met, which is defined as the error between the modal responses obtained in two consecutive iterations:

(11) $$\sum_{k=1}^{K}\frac{{\left\|{\hat{u}}_{k}^{n+1}-{\hat{u}}_{k}^{n}\right\|}_{2}^{2}}{{\left\|{\hat{u}}_{k}^{n}\right\|}_{2}^{2}}<\varepsilon$$

where ε is the selected limit of convergence criteria.

### 2.2. VMD Optimized with WOA

A new heuristic optimization algorithm called the whale optimization algorithm (WOA) was proposed because Mirjalili et al. were inspired by the social behavior of humpback whales [16]. Since its launch, WOA has gained tremendous appeal in the academic community and has been widely applied in various fields. WOA can be divided into three different stages in mathematics.

Prey encirclement: first identify the prey. After successfully identifying the prey’s position, use the following formula to mathematically represent it:

(12) $$D=\left|C.X^{*}\left(t\right)-X\left(t\right)\right|$$

(13) $$X\left(t+1\right)=X^{*}\left(t\right)-A.D$$

X represents the position vector of the whale, $X^{*}$ represents the optimal position of the captured target, t represents the current iteration, and *A* and *C* represent the coefficient vectors. The iterative update of whale position to obtain the optimal solution is represented by the following equation:

(14) A=2a−a

(15) C=2⋅r

Bubble net attack method: “Bubble attack” is a special catching method unique to humpback whales. The spiral updating method of the tight encirclement mechanism and position can be used to analyze the bubble web of cetaceans. Assuming that these two behaviors have an equal probability of 50% for whale position updates, the digital model is as follows:

(16) $$X=\left(t+1\right)=\left\{\begin{array}{c} X^{*}\left(t\right)-A.D\;\;if\;\;0\leq p<0.5\\ D^{\prime }\cdot e^{bl}\cdot \cos\left(2\pi l\right)+X^{*}\left(t\right)\;\;if\;\;1\geq p\geq 0.5 \end{array}\right.$$

(17) $$D=\left|C.X_{random}\left(t\right)-X\left(t\right)\right|$$

(18) $$X\left(t+1\right)=X_{random}\left(t\right)-A\cdot D$$

**Figure 2:**
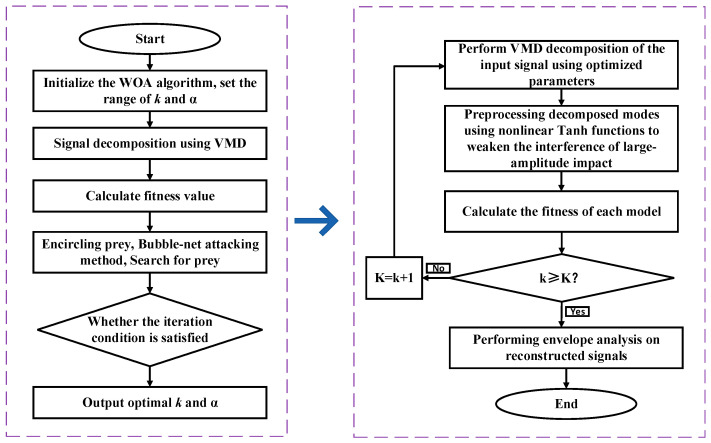
Flow chart of the VMD optimized with WOA.

The steps of the suggested technique are depicted in Figure 2.

### 2.3. Impulse Response Function

The discrete time series was denoted as follows:

Impact force $f_{s}\left(n\right)\begin{array}{cc} & n=0,1,\cdots ,N-1 \end{array}$.

Acceleration $a_{s}\left(n\right)\begin{array}{cc} & n=0,1,\cdots ,N-1 \end{array}$.

Response function $h\left(n\right)\begin{array}{cc} & n=0,1,\cdots ,N-1 \end{array}$.

The transfer function can be calculated by Equation (19).

(19) $$H(k)=\frac{A(k)}{F(k)}$$

(20) $$A(k)=FFT(a_{s}(n))$$

(21) h(n)=IFFT(H(k))

where *FFT* and *IFFT* are the fast Fourier Transform and the inverse *FFT*.

### 2.4. Reconstruction Based on Reconstruction

Research on load identification is very valuable in engineering. Load identification is a typical inverse problem with the nonuniqueness of the solution. In the two previous chapters, the response of the piston slap and the transfer function between the input and output were determined. In this section, we can obtain the slap force by using the deconvolution method of Equation (22).

#### 2.4.1. Theory of Deconvolution

The vibration discrete time series, response function series, and slap force series were denoted as follows:

$$a_{s}=\left(a_{s}\left(1\right),\;a_{s}\left(2\right),\;\cdots ,\;a_{s}\left(l\right)\right)\begin{array}{cc} & l\in Z \end{array}$$

$$h=\left(h\left(1\right),\;h\left(2\right),\;\cdots ,\;h\left(m\right)\right)\begin{array}{cc} & m\in Z \end{array}$$

$$f_{s}=\left(f_{s}\left(1\right),\;f_{s}\left(2\right),\;\cdots ,\;f_{s}\left(k\right)\right)\begin{array}{cc} & k\in Z \end{array}$$

The discrete convolution equation was obtained using Equation (22).

In Equation (15), the slap force series *f_s_*(*n*) is an unknown series needed to be estimated. According to the convolution algorithm, we can change Equation (22) into a matrix multiplication as Equation (23).

(22) $$a_{s}\left(n\right)=f_{s}\left(n\right)*h\left(n\right),n=1,2,\cdots ,l$$

In Equation (15), the slap force series $f_{s}\left(n\right)$ is an unknown series needed to be estimated. According to the convolution algorithm, we can change Equation (22) into a matrix multiplication as Equation (23).

(23) $$a_{s}=f_{s}\cdot H$$

where H is an N by M matrix. No inverse of the matrix *H* exists. However, a pseudo-inverse matrix denoted as H can be obtained to address this problem. Thus, the slap force can be acquired using the following equation.

(24) $$f_{s}=a_{s}\cdot H^{+}$$

This way, the convolution operation is converted into solving the pseudo-inverse matrix.

#### 2.4.2. Regularization of Ill-Posed Matrix

The pseudo-inverse matrix in Equation (24), often called the discrete ill-posed problem, was solved. The matrix H may be ill-conditioned or singular and may yield a nonunique solution that can be judged by the condition number of the matrix H (the ratio of the largest singular value to the smallest value). Tikhonov regularization is the most commonly used method for regularizing ill-posed problems [17,18]. The standard approach to solving an underdetermined system of linear equations, such as Equation (23), is known as linear least squares. It seeks to minimize the residual regularization.

(25) $${\left\|\left.f_{s}\cdot H-a_{s}\right\|\right.}^{2}$$

where ||·|| is the Euclidean norm. Tikhonov regularization determines a useful approximation of ${\hat{f}}_{s}$ to provide preference to a particular solution with desirable properties by using a penalized least-squares problem of the form.

(26) $${\left\|f_{s}\cdot H-a_{s}\right\|}^{2}+\beta {\left\|f_{s}\right\|}^{2}$$

The scalar β>0 is the regularization parameter. The key to this approach is making an appropriate choice of regulation parameters β to find a compromise between the data fitting effect and minimum norm solution. The generalized cross-validation (GCV) method is a very popular and successful error-free method for choosing the regularization parameter [19,20]. It does not require knowledge of noise variance. The SVD of H is presented by Equation (10).

(27) $$H=V\Lambda W^{T}$$

where $V=\left[V_{1},V_{2},\cdots ,V_{k}\right]\in R^{k\times k}$ and $W=\left[W_{1},W_{2},\cdots ,W_{l}\right]\in R^{l\times l}$ are the orthogonal matrices that satisfy the important relations $WW^{T}=I$ and $VV^{T}=I$ ($W^{T}$ is the Hermite transpose), and Λ is a (always rectangular) diagonal matrix whose diagonal entries σj  are the singular values. Equation (27) is substituted into Equation (24), which is rewritten as follows:

(28) $$f_{s}=a_{s}W\Lambda^{+}V^{T}=a_{s}\sum_{i=1}^{MP}\frac{1}{\sigma_{i}}W_{i}V_{i}^{T}$$

(29) $$\Lambda ={\left[\begin{array}{ccccccc} \sigma_{1} & 0 & 0 & \cdots & 0 & 0 & \cdots \\ 0 & \sigma_{2} & 0 & \cdots & 0 & 0 & \cdots \\ 0 & 0 & \sigma_{3} & \cdots & 0 & 0 & \cdots \\ \vdots & \vdots & \vdots & \ddots & \vdots & \vdots & \vdots \\ 0 & 0 & 0 & \cdots & \sigma_{k} & 0 & \cdots \end{array}\begin{array}{c} 0\\ 0\\ 0\\ \vdots \\ 0 \end{array}\right]}_{k\times l}\in R^{k\times l}$$

We can obtain the regulation solution using the *GCV* method.

(30) $$f_{s}=a_{s}W\Lambda^{+}V^{T}=a_{s}\sum_{i=1}^{MP}\frac{\sigma_{i}}{{\sigma_{i}}^{2}+\beta }W_{i}{V_{i}}^{T}$$

The *GCV* function was denoted as follows:

(31) $$GCV\left(\beta \right)=\frac{\left(1/k\right){\left\|\left(I-B(\beta )\right)P\right\|}^{2}}{{\left[\left(1/k\right)\mathrm{Tr}(I-B(\beta ))\right]}^{2}}$$

where $B\left(\beta \right)=\psi \left(\psi^{T}\psi +\beta I\right)\psi^{T}$ and Tr is the trace of the matrix. We selected the best regularization parameter when the $GCV\left(\beta \right)$ reached the maximum value.

## 3. Testing Setup

In our study, the measurements were carried out on an in-line four-stroke four-cylinder diesel engine. The parameters are shown in Table 1. Minimizing the effect of additive noise is crucial. The best way to minimize the effect is to carefully choose the location of the vibration transducer. The cylinder block, which mainly concerns the piston slap, exhibits a satisfactory signal-to-noise ratio. Transducers were placed on the thrust side, and transversal acceleration was considered because it represents the most significant direction for studying the piston slap phenomenon [21,22].

**Figure 3:**
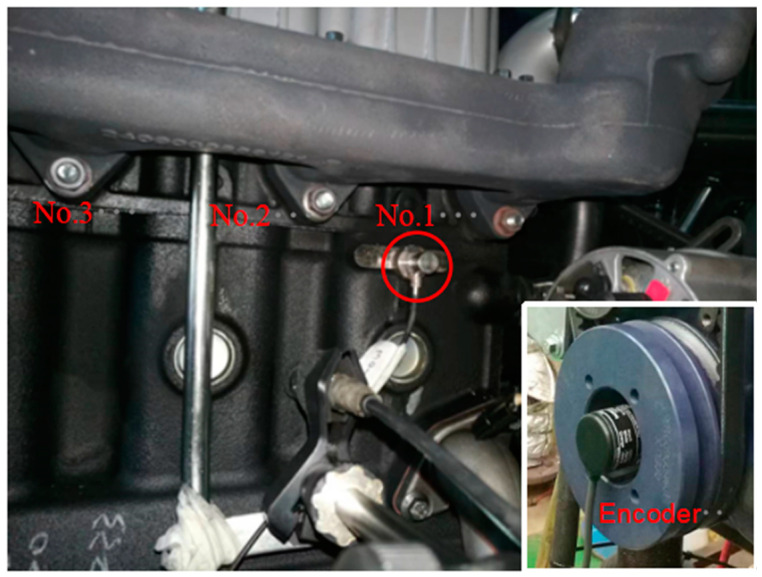
The location of transducers on cylinder block.

One accelerometer sensor was placed on the thrust side surface of the first cylinder block, which is at the centerline of the cylinder and near the top dead center. Figure 3 shows the location of the accelerometer. The first cylinder is equipped with a pressure sensor in the preheating plug hole and an encoder installed at the end of the crankshaft to obtain the angular position of the crankshaft.

The phenomenon of piston slap occurs when the piston collides with the internal wall of the cylinder. Burning and piston slap occur simultaneously in the process of gas combustion in a cylinder. At this time, the valves corresponding to the cylinder being tested are closed. Thus, its vibration is neglected. Moreover, the gears and the engine accessory system are far from the position. The vibration corresponding to it is also neglected. Therefore, the highest SNR signals obtained at this location consist of only two signals produced by combustion and slap.

**Figure 4:**
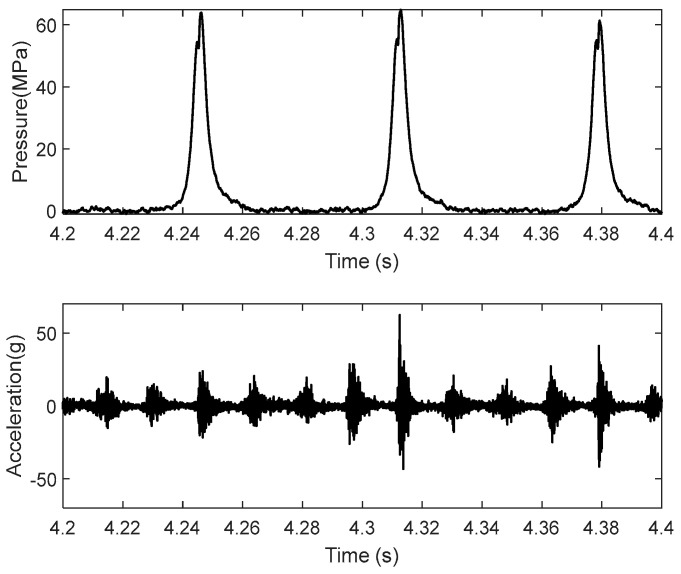
The sequence of signal at 1800 rpm and 160 Nm.

All signals were recorded with a sampling frequency of 32 kHz. The discrete time series is denoted as $a\left(n\right)$ and $p\left(n\right),\;n=1,2,3\ldots ,N$, representing the vibration and the combustion pressure discrete signal series, respectively. N is the number of total sample points. Figure 4 shows a recorder in the case of 1800 rpm and 160 Nm. The signals perform strong transient characteristics. One cycle of the signals, which was synchronous to the rotation speed of the crankshaft, was truncated in the angular window [−20, 60] of the crankshaft, as shown in Figure 4.

## 4. Piston Slap Response Separation

**Figure 5:**
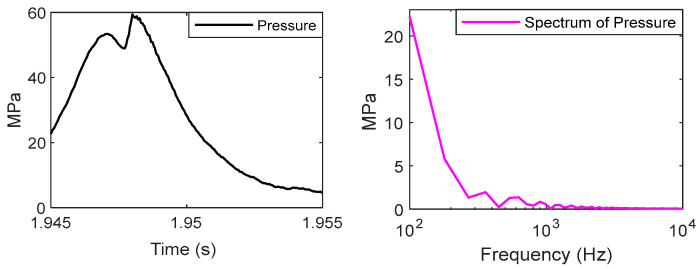
The cylinder pressure and the spectrum at 1800 rpm and 160 Nm.

**Figure 6:**
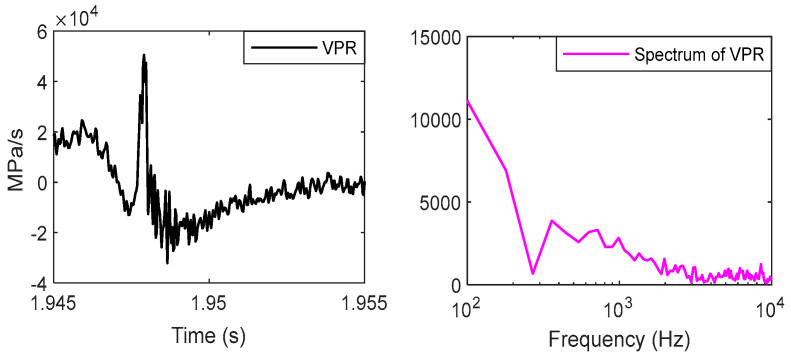
The velocity of pressure rise and the spectrum at 1800 rpm and 160 N.

A truncated signal a(n) which occurs within the range of −20 °CA–80 °CA is decomposed into four IMF components, and the corresponding frequency characteristics of it are shown in the same figure. The central frequencies of IMFs are 1000 Hz, 3500 Hz, 4500 Hz, and 8000 Hz. The spectrum of combustion pressure and velocity of pressure rise (VPR) are shown in Figure 5 and Figure 6. Obviously, the energy of them is mainly distributed in the range of 0–1000 Hz.

**Figure 7:**
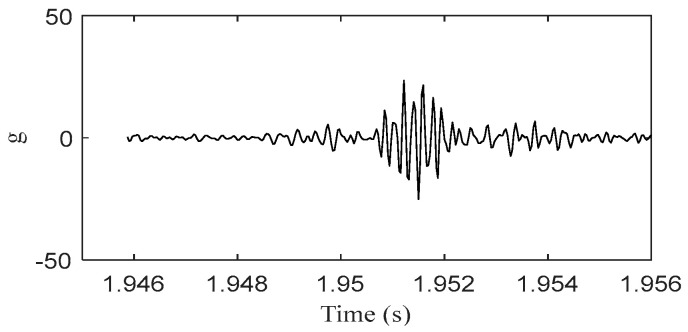
The response of piston slap at 1800 rpm and 160 Nm.

**Figure 8:**
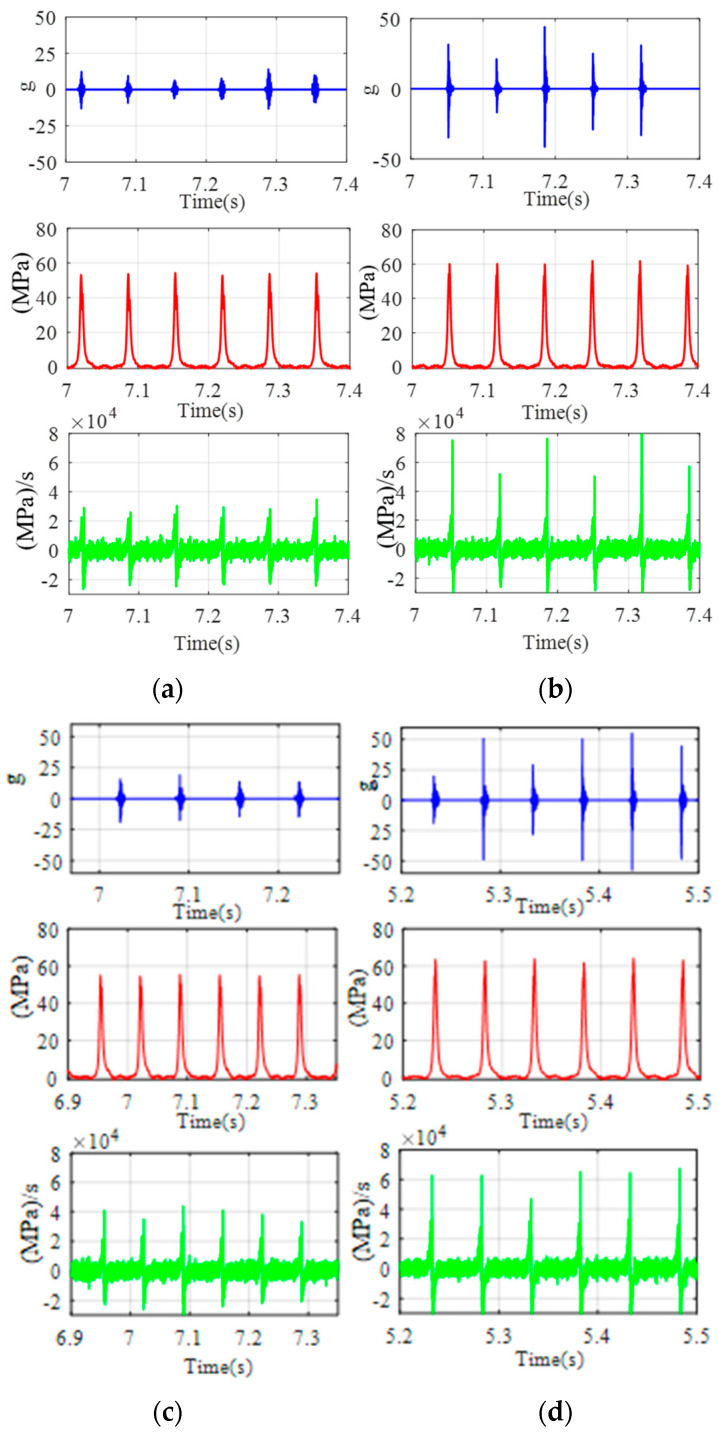
The vibration of slap force under different engine operating conditions: (**a**) 1800 rpm/80 Nm/−11 °CA; (**b**) 1800 rpm/80 Nm/−17 °CA; (**c**) 1800 rpm/160 Nm/−11 °CA; (**d**) 2400 rpm/160 Nm/−11 °CA.

Two articles [22,23] have studied the relationship between combustion pressure and the vibration of cylinder blocks (Figure 7 and Figure 8). 

Pressure has most of its energy concentrated at a low frequency, below a few hundred hertz, and its vibration is found to have little energy there due to the high rigidity of the engine block.

The signals of block vibration and cylinder pressure were analyzed using a CWT [23], and the results show that the maximum heat release rate has a strong correlation with the magnitude of the vibrations. A specific bandwidth, vibration signals of 0.3~1.5 kHz, was affected by variation in the heat release rate. The vibrations excited by combustion lasted over 50 °CA.

Based on the analysis of these data, we can draw the following conclusions: 

(1) The energy distribution of combustion excitation is in the range of 0–2000 Hz, and the first component, IMF1, is produced by combustion pressure and piston slap.

(2) The spectrum of the other components, IMF2, IMF3, and IMF4, is higher than the excitation of combustion, so they are produced only by the piston slap.

**Figure 9:**
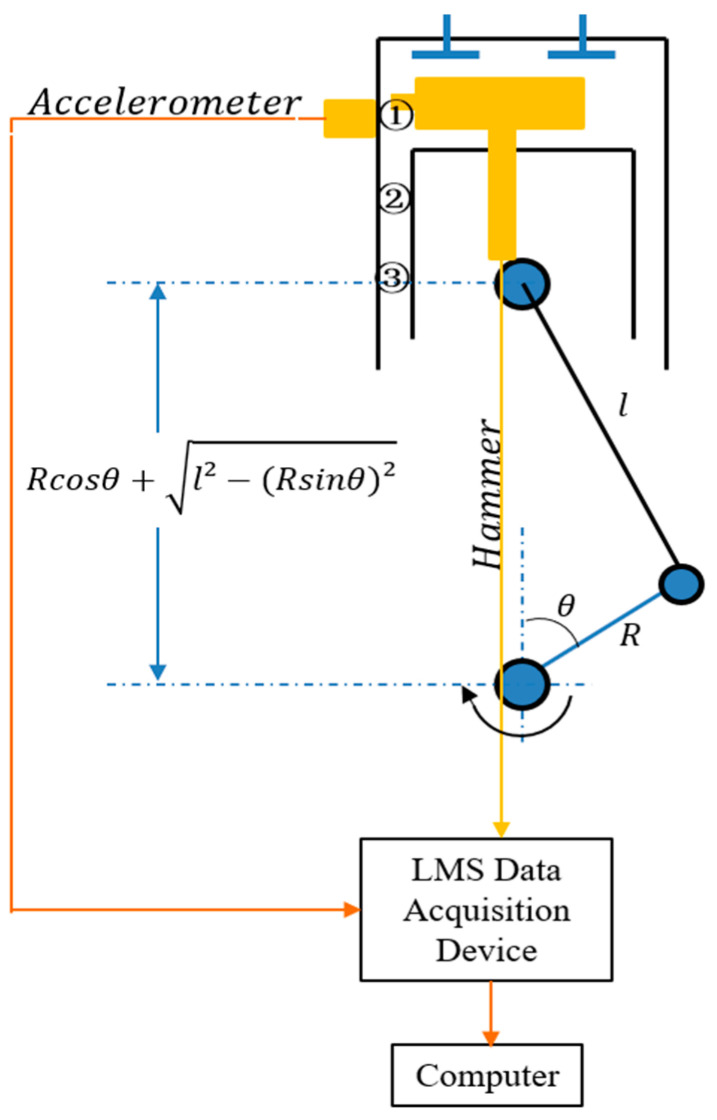
Experimental setup to measure the response at impact points.

Due to the fact that the energy of IMF1 is a small part of the total energy of the vibration, this component is ignored, and the other components are selected to construct the response of the piston slap, as shown in Figure 9. Using this method, the characteristics of slap force response are analyzed in detail under different loads; for example, Figure 8 shows the slap force response on four different kinds loads. Firstly, at the same speed and the same torque, the large injection advance angle will cause an increase in the velocity of pressure rise and lead to the increase in slap force response, shown in Figure 8a,b. Secondly, at the same speed and the same injection advance angle, the vibration of slap force increases with increasing torque, shown in Figure 8a,c. If we compare Figure 8b,c, we can reach the conclusion that a lower torque with a large injection advance angle will cause a larger vibration of piston slap. Thirdly, at the same torque and the same injection advance angle, the vibration of slap force increases with increasing speed. From the above analysis, it can be seen that the main factors affecting the vibration of slap are torque, speed, and velocity of pressure rate.

## 5. Impulse Response Function Testing

The method for reconstructing the slap force strongly depends on the nature of the input–output relationship between the slap force and its resulting vibration on the cylinder block. For simplicity, a linear relationship is assumed in the vibration transfer path in the engine block. A hammer test is the most convenient and effective way of obtaining the response function of the block.

**Figure 10:**
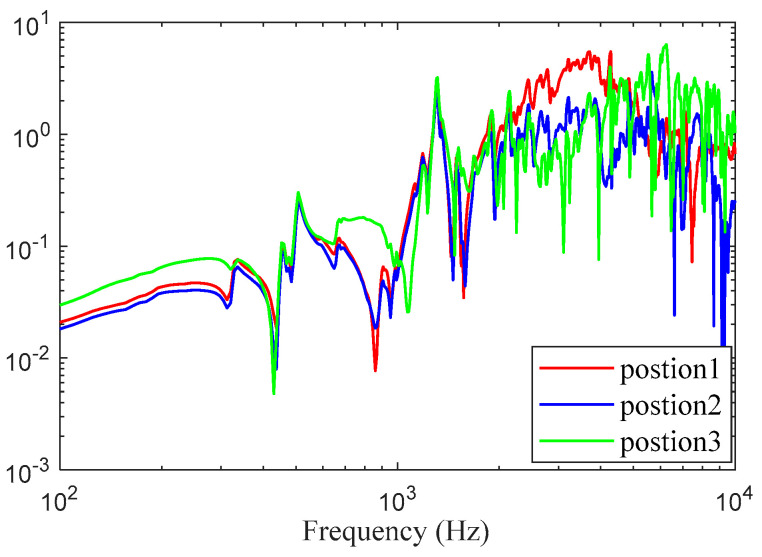
Frequency response function curve.

**Figure 11:**
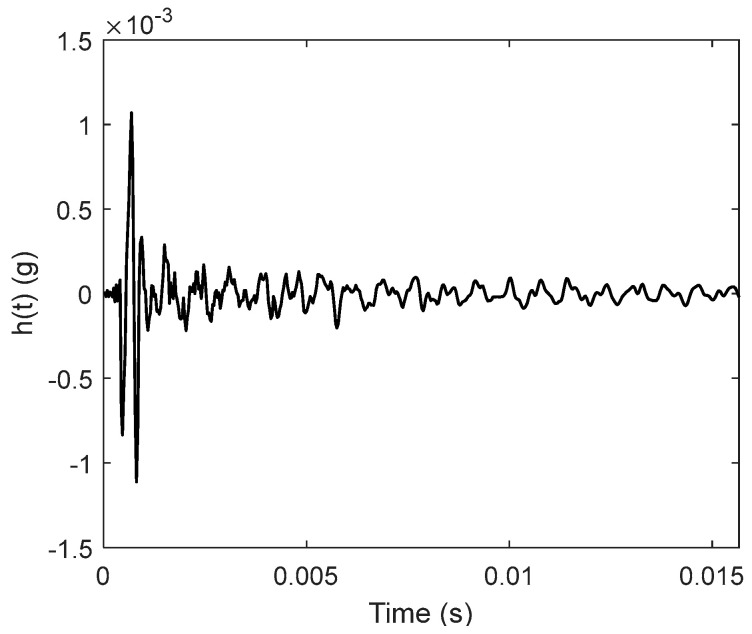
Impulse response function curve.

One of the most important things in the test is selecting the impact position on the cylinder. Piston slaps always occur in the power stroke and on the thrust side of the cylinder bore because the maximum pressure occurs in this process with the rapid rise in pressure (Figure 10 and Figure 11).

**Figure 12:**
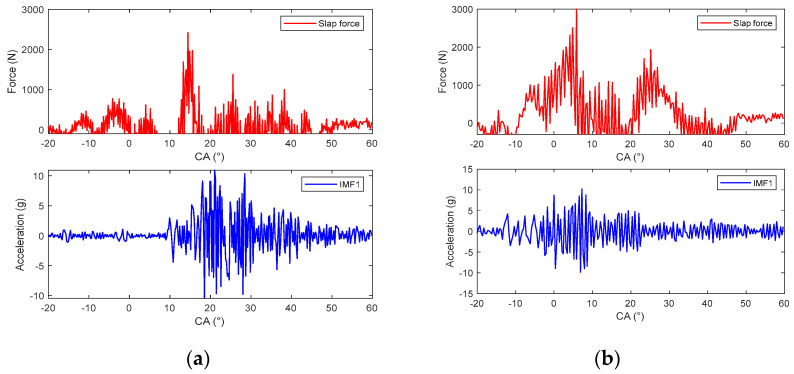
Reconstruction of flapping force with different parameters: (**a**) 1200 rpm and 10 Nm; (**b**) 1800 rpm and 10 Nm.

**Figure 13:**
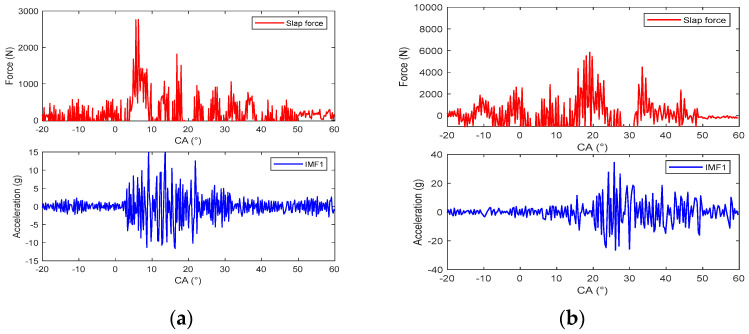
Reconstruction of flapping force with different parameters: (**a**) 1200 rpm and 160 Nm; (**b**) 1800 rpm and 160 Nm.

**Figure 14:**
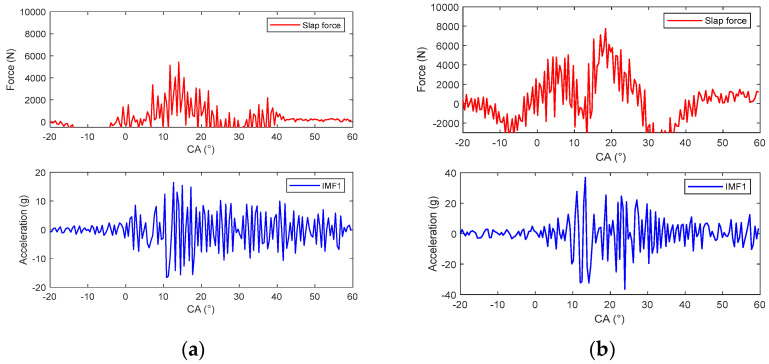
Reconstruction of flapping force with different parameters: (**a**) 2400 rpm and 10 Nm; (**b**) 2400 rpm and 160 Nm.

**Figure 15:**
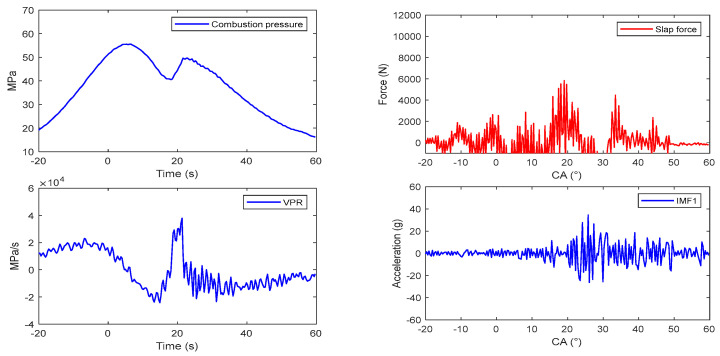
Slap force reconstruction at 1800 rpm and 160 Nm with injection advance angle of 11 °CA.

**Figure 16:**
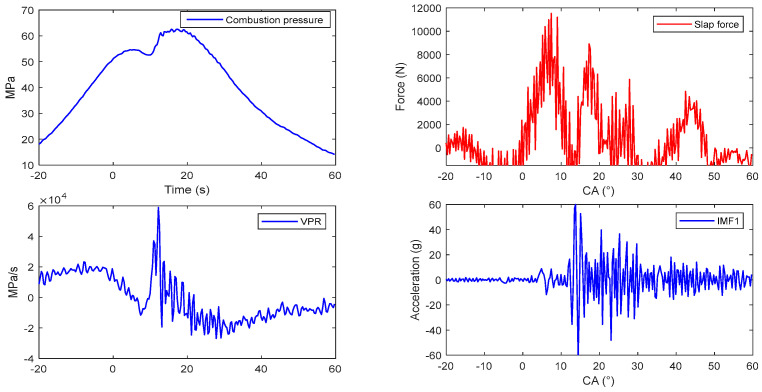
Slap force reconstruction at 1800 rpm and 160 Nm with injection advance angle of 18 °CA.

According to the force analysis of the piston in the cylinder liner using the formula $F_{s}=F_{g}tan\theta$ (ignoring the friction between the piston and cylinder bore), the maximum slap force must occur in this period. Figure 12, Figure 13, Figure 14, Figure 15 and Figure 16 display the vibration response distribution from −20 °CA to 60 °CA. Thus, three positions were selected on the thrust side. The corresponding angles of crank are 15 °CA, 35 °CA, and 55 °CA. The distance between the hammer impact location and crank axis can be determined using formula $Rcos\theta +\sqrt[{}]{l^{2}-{\left(Rsin\theta \right)}^{2}}$, as shown in Figure 8. The position of the accelerometer is shown in Figure 4. The vibration frequency response is up to 10,000 Hz. The useful frequency range of the transducer and the hammer must reach the limit. A test was conducted using a Siemens LMS Test-lab system. The selected accelerometer and hammer are Kistler products. All signals were sampled at 32 kHz.

Figure 10 shows the frequency response function between the input and output. The vibration energy is concentrated above 3000 Hz. The impulse response function was calculated using Equation (6) and is depicted in Figure 11. The vibration decays rapidly to zero, indicating that a large internal damping occurs in the cylinder body.

## 6. Result Analysis of Slap Force Reconstruction

Using the deconvolution method, the slap forces were obtained at six different engine load conditions: (1) 1200 rpm/10 Nm and 120 Nm, (2) 1800 rpm/10 Nm and 160 Nm, and (3) 2400 rpm/10 Nm and 160 Nm, as shown in Figure 12, Figure 13, Figure 14, Figure 15 and Figure 16. All of these engine load conditions have the same injection advance angle of 11 °CA.

The calculation results reveal that, under the same load, the slap force increases with the rotational speed. Similarly, at the same speed, the slap force increases with the engine torque, because the greater torque requires a higher gas pressure. The relationship between the cylinder pressure and slap force can be expressed by the formula $F_{s}=F_{g}tan\theta$, so $F_{s}$ increases with Fg (here, ignoring the friction between the piston and cylinder bore).

In all the obtained results, the piston slaps occur within the range of 0 °CA–30 °CA after the top dead center of the power stroke. The slap force waveforms consistently exhibit two peaks, indicated by the dotted circles in Figure 12, which are attributed to the fact that, within this range, the combustion of the combustible gases is the most intense. The piston slap did not happen at the moment of maximum combustion pressure but at the maximum velocity of pressure rise (VPR). Meanwhile, the greater injection advance angle is always accompanied by greater slap force. The delay period exerts a great influence in the diesel engine combustion phenomenon. The longer the delay, the more rapidly and higher the temperature rises because more fuel will be present in the cylinder before the rate of burning comes under control, which causes rougher running and stronger diesel knock.

## 7. Conclusions

A novel method is presented for the estimation of piston slap force in this article. This method can optimize the $\left[Κ,\alpha \right]$ parameter combination of the traditional VMD method using WOA, which improves the intelligence of the algorithm and simplifies the selection process. The vibration signal on the block surface can be decomposed into multiple IMF components by the WOA-VMD method. The slap force response can be obtained using the correlation coefficient between IMF1 and cylinder pressure. 

The transfer function between the incentive and vibration response points is obtained using the percussion test. Subsequently, the slap force was calculated using the deconvolution method. In this process, converting the convolution operation into solving the pseudo-inverse transfer matrix is the key step. Sometimes, the transfer matrix is ill-conditioned. To address this issue, Tikhonov regularization is commonly employed to overcome this limitation and improve the accuracy of the calculations.

This method provides a valuable means of evaluating the angular distribution of the slap force, identifying the shock positions on the piston liner, and determining the peak value of the waveform. All of this work can help us to study the vibration characteristics of pistons and enhance the structural design of engines. This method can be extended to various engine configurations and types, including gasoline and hybrid engines, to validate its versatility and robustness. The findings can be incorporated into predictive maintenance frameworks to preemptively address potential issues in engine components, thereby reducing downtime and maintenance costs.

## Figures and Tables

**Table 1:** Engine specification.

Engine Specification	
Displacement	3.0 L
Rated Power/RPM	38 kW@2400 rpm
Max-Torque/RPM	180 Nm@1500–2000 rpm
Fuel system	common rail
Bore × Stroke (mm)	93 × 112
Compression ratio	17
Intake Type	natural
Max injection pressure	15 MPa
Firing order	1-3-4-2

## Data Availability

The data that support the findings of this study are available from the corresponding author, zhzz18@126.com, upon reasonable request.
